# Methanol extract of *Ligusticum chuanxiong* Hort. Rhizome ameliorates bilateral common carotid artery stenosis-induced cognitive deficit in mice by altering microglia and astrocyte activation

**DOI:** 10.3389/fphar.2024.1329895

**Published:** 2024-03-14

**Authors:** Sehyun Lim, Chiyeon Lim, Suin Cho

**Affiliations:** ^1^ School of Public Health, Far East University, Eumseong, Republic of Korea; ^2^ Department of Radiology, Massachusetts General Hospital and Harvard Medical School, Charlestown, MA, United States; ^3^ College of Medicine, Dongguk University, Goyang, Republic of Korea; ^4^ School of Korean Medicine, Yangsan Campus of Pusan National University, Yangsan, Republic of Korea

**Keywords:** Chuanxiong Rhizoma, *Conioselinum anthriscoides* “Chuanxiong”, aging, dementia, cognitive performance, neurodegenerative disorders

## Abstract

In traditional Asian medicine, *Ligusticum chuanxiong* Hort also known as *Conioselinum anthriscoides* “Chuanxiong”, is mainly used for improving blood circulation or for analgesic and anti-inflammatory purposes, but they also have a long history of use for pain disorders in the head and face, such as headache. Despite the possibility that the plant is effective for diseases such as cerebral infarction and vascular dementia (VaD), the mechanism of action is not well understood. To determine if the dried rhizomes of *L. chuanxiong* (Chuanxiong Rhizoma, CR) methanol extract (CRex) has activity in a VaD mice model. Through network analysis, we confirm that CR is effective in cerebrovascular diseases. In mice, we induce cognitive impairment, similar to VaD in humans, by chronically reducing the cerebral blood flow by performing bilateral common carotid artery stenosis (BCAS) and administering CRex for 6 weeks. We measure behavioral changes due to cognitive function impairment and use immunofluorescence staining to confirm if CRex can inhibit the activation of astrocytes and microglia involved in the inflammatory response in the brain. We quantify proteins involved in the mechanism, such as mitogen-activated protein kinases (MAPK), in the hippocampus and surrounding white matter, and analyze gene expression and protein interaction networks through RNA sequencing to interpret the results of the study. CRex administration rescued cognitive impairment relating to a novel object and inhibited the activation of astrocytes and microglia. Western blotting analysis revealed that CRex regulated the changes in protein expression involved in MAPK signaling such as extracellular signal-regulated kinase (ERK) and p38 mitogen-activated protein kinase (p38). The administration of CRex suppressed approximately 44% of the pathological changes in gene expression caused by BCAS. CRex extract effectively inhibited cognitive impairment caused by BCAS, and the mechanism through which this occurred is inhibited activation of astrocytes and microglia.

## 1 Introduction

Cerebrovascular disorders (CVDs) are caused mainly by a decrease in blood flow to the brain that leads to hypoxia in the central nervous system, resulting in endothelial dysfunction, cell death with an increase in vascular inflammatory factors, and damage to the hippocampus with a decrease in microcirculation (Venkat et al., 2015). Vascular dementia (VaD) is a vascular cognitive disorder, a clinical cognitive impairment of cerebrovascular origin, and is the second most common cause of dementia after Alzheimer’s disease (AD) (O'Brien and Thomas, 2015). The pathophysiology of VaD is diverse and has not yet been clearly established; in addition, various subtypes exist. The close relationship between VaD and AD is known, as vascular damage coexists with indicators of AD in many patients (Craft, 2009; O'Brien and Thomas, 2015). Degeneration of neuronal cells in the brain causes AD. VaD is caused by the blockage or narrowing of cerebral blood vessels, which results in poor blood supply to the brain and damage to brain tissue (Kalaria et al., 2008; O’Brien and Thomas, 2015; Venkat et al., 2015; Zhang et al., 2016). High blood pressure, diabetes, and hyperlipidemia are common risk factors for AD and VaD; and both diseases are associated with reduced acetylcholine levels, thus share treatment drugs. However, unlike AD, VaD symptoms appear relatively suddenly and worsen in a clear stepwise manner. Along with cognitive impairment such as memory decline, the neurological symptoms, such as speech impairment, facial paralysis, hemiparesis, balance impairment, and gait impairment have also been observed (Baskys and Hou, 2007; Allan et al., 2011; Zhang et al., 2016).

VaD accounts for approximately 15%–20% of all dementia cases in North America and Europe and approximately 30% of all dementia cases in Asia and developing countries (Wolters and Ikram, 2019). VaD has been reported to occur in more than a few stroke patients, and as it is dementia originating from the cerebrovascular system, it can damage various areas of the central nervous system. It is reported that the burden of care is higher than that of patients with AD as they develop disabilities in various areas, decreased daily living ability, and progress to severe symptoms (Allan et al., 2011). The pathogenesis of VaD involves a variety of processes including reduced cerebral hemodynamics and potential therapeutic targets, include immune response, apoptosis, and extra- and intracellular signaling molecules (Baskys and Hou, 2007; Craft, 2009; Allan et al., 2011; Li et al., 2021). However, unlike AD, there is no approved drug that is pharmacologically effective. This is due to the special nature of VaD, which has a complex presentation; for this reason, the current primary treatment for VaD is to control vascular risk factors (Baskys and Hou, 2007). Therefore, there is a need for research into drugs for VaD treatment, and there is a view that drugs should be able to simultaneously act on multiple targets; drugs altering inflammation, apoptosis, and cell adhesion are expected to be developed as effective treatments for VaD in the future (Brucki et al., 2011). Thus, it appears that we should also pay attention to approaches taken in traditional Asian medicine.

Currently, herbal medicines are used widely in the treatment of vascular dementia in Asian countries, especially Korea and China. Based on a meta-analysis of herbal medicine treatments for VaD, the administration of herbal medicines to treat VaD should be relatively safe and effective (Qin et al., 2013; Zeng et al., 2015; Xu et al., 2018). Therefore, attempts are being made to search for and develop treatments for VaD using a single drug, a specific compound, or a prescription composed of specific drugs (Ho et al., 2011; May et al., 2012).

In this study, we attempt to confirm whether symptoms of VaD in a mouse model can be ameliorated by Chuanxiong Rhizoma (CR), the dried rhizomes of *Ligusticum chuanxiong* Hort. *L. chuanxiong* is a perennial herb in the Umbelliferae family. The herb is known by the scientific name *L. chuanxiong* in Korea and China, where it is primarily used and the official scientific name is *Conioselinum anthriscoides* “Chuanxiong”, thus, *L. chuanxiong* is a synonym for *C. anthriscoides* “Chuanxiong”. Since the rhizome of this plant was first described in *Sheng-Nong’s Classic of Materia Medica*, it has been considered as ‘*qi* medicine in the blood’ in traditional Asian medicine because of its excellent efficacy in stimulating blood circulation and warding off winds; thus, it is used widely for various pain and gynecological diseases. CR is one of the major cardiovascular protective herbal medicines used clinically and it is a popular pharmaceutical, commonly used in prescriptions for the treatment of atherosclerosis, ischemic stroke, angina pectoris, and hypertension. It also has antioxidant, neuroprotective, and anti-inflammatory activities (Li et al., 2012; Gao et al., 2017; Yang et al., 2020). A study investigating *Caenorhabditis elegans* has reported that ethyl alcohol extracts obtained from the leaves and rhizomes of CR could be effectively used for treating AD, as they inhibit oxidative damage to *C. elegans* (Qin et al., 2022). However, research demonstrating that CR is active against various types of dementia, including VaD is still lacking.

CR has been reported as one of the most frequently used medicinal herbs of the components of Asian traditional medicine prescriptions that are used to treat patients with VaD. In addition, among the various compounds, tetramethylpyrazine and cnidiuml actone are known to alter ischemic brain damage by improving the blood flow in micro-vessels to the brain; thus, clinical studies are in progress to evaluate their effects on patients’ recovery from cerebrovascular accidents (Zheng et al., 2018).

Because herbal medicines contain many metabolites, it is difficult to clearly identify which of these metabolites demonstrates specific activity. In addition, as it cannot be said that one compound exhibits only one activity, we confirmed the evidence that CR can be effectively used for cerebrovascular disease through network analysis and in experimental mice with VaD induced by bilateral common carotid artery stenosis (BCAS). Rats and mice are mainly used as rodent models for VaD. The chronic cerebral hypoperfusion model involving rats is associated with disadvantages, such as damage to the optic nerve and difficulties in genetic evaluation. The ligation of both common carotid arteries in mice, similar to that in rats is associated with a disadvantage of causing death in a short time period. To overcome these limitations, a more appropriate animal model is required. We overcame the above shortcomings by using a mouse model of chronic cerebral hypoperfusion induced by BCAS (Washida et al., 2019). Subsequently, it was confirmed whether the damage caused in the brain could be suppressed by the administration of CR methanol extract (CRex). To confirm the pathological changes caused by BCAS and their inhibition by CRex administration, behavioral changes were observed, changes in brain tissue protein expression were confirmed through Western blotting, the degree of damage and inhibition was confirmed by brain tissue staining, and gene expression changes were measured.

## 2 Materials and methods

### 2.1 Network analysis

Network pharmacology research is often conducted on Asian traditional medicine databases; a representative database is the Traditional Chinese Medicine Systems Pharmacology Database and Analysis Platform (TCMSP) (Ru et al., 2014), which is mainly used in papers on Chinese medicine and has been used for performed many network pharmacology studies. In this study, the analysis was conducted using the TCMSP database; the components expected to be active were selected and then the absorption, distribution, metabolism, and excretion (ADME) characteristics of each component were determined, and pharmacokinetically active compounds with potential potency were selected.

The ADME variables considered include oral bioavailability (OB), which indicates the ability of an orally administered drug to be delivered to the body; drug likeness (DL), which helps to optimize pharmacokinetic properties by estimating how many potential compounds are present, such as known drugs; effect in Caco-2 cells, which can indicate drug absorption in the gastrointestinal tract; and blood–brain barrier (BBB) permeability, which acts as an anatomical barrier to restrict the passage of proteins into the central nervous system. When determining the DL property of bioactive molecules as therapeutic agents, high OB is often used as a crucial indicator. The DL level of the compounds, which is 0.18, is used as a selection criterion for DL compounds in herbal medicine. Herein, the compounds’ transport rates (nm/s) in Caco-2 monolayers were used to represent the intestinal epithelial permeability in TCMSP. The compounds with BBB ≤ −0.3 were considered as non-penetrating (BBB−), from −0.3 to +0.3 were considered moderately penetrating (BBB±), and ≥0.3 were considered strongly penetrating (BBB+). For each variable satisfying OB ≥ 30%, DL ≥ 0.18, and Caco-2 ≥ 0, which are recommended as the minimum values of variables available in TCMSP and ingredients satisfying BBB ≥ −0.3 (i.e., those that can pass BBB) are selected in this study (Jiwa et al., 2010). For the compounds selected using above method, the diseases for which each compound could be applied were checked in the TCMSP database to construct a compound-target protein and target protein-disease networks. The Cytoscape software (ver. 3.10.0) was used to visualize these networks.

### 2.2 Reagents

Paraformaldehyde (4% PFA) was from Sigma-Aldrich Co. (St. Louis, MO, USA), phosphate-buffered saline (PBS) was from Bio Basic Inc. (Markham, Ontario, Canada), sucrose was manufactured by Junsei Chemical Co. Ltd (Chuo-ku, Tokyo, Japan), saline solution was purchased from Choongwae Pharmaceutical (Seocho-gu, Seoul, Korea). The optimal cutting temperature (OCT) compound was from Scigen Scientific Inc. (Gardena, CA, USA), Pro-PREP was purchased from iNtRON (Seongnam-si, Gyeonggi-do, Korea). Rabbit-anti goat IgG-HRP was from Santa Cruz Biotechnology Inc. (Dallas, TX, USA), PAP pen, Envision Kit DAKO K5007 were purchased from DAKO (Santa Clara, CA, USA). BCA reagent and albumin standard were purchased from Thermo Fisher Scientific (Waltham, MA, USA), and Tris and glycine were purchased from Generay Biotech (Shanghai, China). Skim milk was purchased from Sigma-Aldrich Co. (St. Louis, MO, USA). Extracellular signal-regulated kinase (ERK), phospho-ERK (*p*-ERK), c-Jun N-terminal kinase (JNK), phospho-JNK (*p*-JNK), p38 mitogen-activated protein kinases (p38), *p*-p38, Wnt family member 3a (Wnt3a), glycogen synthase kinase 3 beta (GSK3β), and *p*-Ser9-GSK3β (*p*-GSK3β) were purchased from Cell Signaling Technology (Boston, MA, USA) and used as the primary antibodies. Beta-actin (β-actin) antibody was obtained from Santa Cruz Biotechnology Inc. (Dallas, TX, USA). Goat anti-rabbit IgG, for use as a secondary antibody, was obtained from Enzo Life Sciences Inc. (Farmingdale, NY, USA), and anti-mouse IgG was purchased from Cell Signaling Technology (Boston, MA, USA). For the ECL solution, West-Q chemiluminescent substrate purchased from GenDEPOT (Katy, TX, USA) and Pierce ECL Plus Western Blotting Substrate purchased from Thermo Fisher Scientific (Waltham, MA, USA) were used.

### 2.3 Herbal material and extraction

The CR used in this experiment was purchased from Kwangmyeongdang Pharmaceutical Co., Ltd. (Ulsan, Korea). It was confirmed that the CR met the quality standards of the Ministry of Food and Drug Safety of Korea (Supplementary Material S1), and a voucher specimen for the CR was refrigerated in the depository of herbal medicine storage room of the Pusan National University Graduate School of Korean Medicine (No. 22CR-129). The weight of the dried medicinal materials used was 1,034 g; the medicinal materials were ground and then immersed in 100% methanol (Deoksan General Science, Seoul, Korea) at 25°C for 2 days. The supernatant was filtered through qualitative filter paper (Whatman, No. 2, Tokyo, Japan) and the filtrate was lyophilized using a rotary evaporator and freeze dryer. The obtained lyophilized CR methanolic extract (CRex) (77.5 g, yield 7.5%), was stored at −20°C.

### 2.4 Animals

**FIGURE 1:**
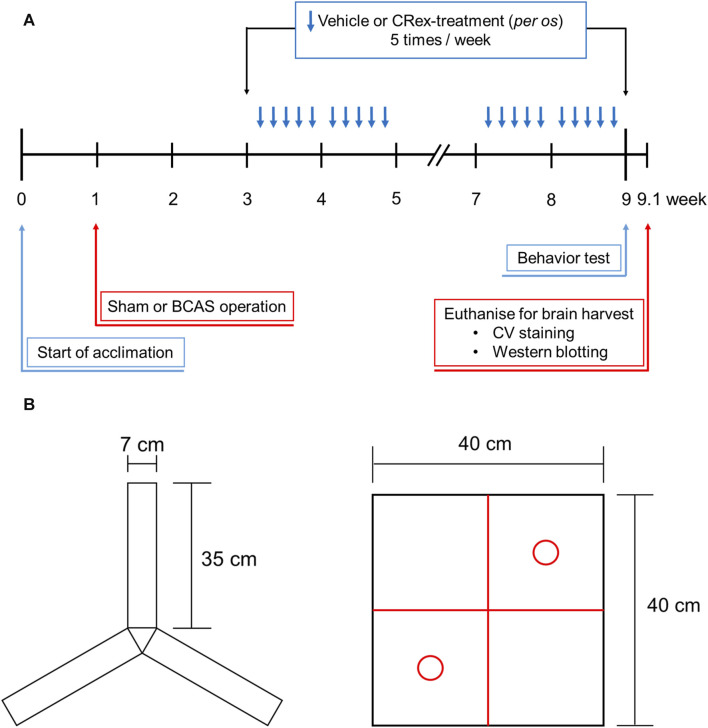
The overall study process and behavioral measures for the Y-maze and open field box. Panel **(A)** shows the timing and specific methods used for each part of the study, and Panel **(B)** shows the two types of objects in the Y-maze that are used to measure the spatial cognitive ability and open field box, which is used for measuring the ability to recognize new objects.

Eight-week-old male C57BL/6 mice purchased from SAMTAKO (Gyeonggi-do, Korea) were used after a 1-week acclimatization period in the animal breeding room. During the adaptation and experiment periods, the environment was maintained at a room temperature of approximately 23°C, humidity of approximately 50%, and a light source-controlled light–dark cycle of 12 h; the animals were permitted to consume food and water autonomously. The management and use of laboratory animals was performed in accordance with the Laboratory Animal Ethics Regulations of the Animal Experimentation Ethics Committee of Pusan National University (PNU-2018-2113). Mice were weighed once a week for 2 months from the age of 8 weeks, with additional measurements on the day of surgery and 2 days after surgery (Figure 1A).

### 2.5 BCAS-induced VD mice model

BCAS is a method of permanently reducing blood flow to both brain hemispheres through narrowing both carotid arteries with micro-coils. For anesthesia, 2.0% isoflurane was supplied with a mixture of 70% N_2_O and 30% O_2_ through the respiratory system during BCAS operation. After anesthesia, the mice were fixed in a supine position on the operating table, and a hot pad attached to the homeothermic system, in conjunction with an animal rectal homeothermic monitoring system (Harvard Instruments, MA, USA), was used to maintain body temperature (36.5°C ± 0.5°C).

The hair on the front neck and the surrounding area of the mouse, which covered the surgical site, was removed using a depilator and clipper, and disinfection was performed with povidone and 70% ethanol (Deoksan General Science, Seoul, Korea) successively. Then, under a stereo microscope, a midline incision was made in the anterior neck, and both carotid arteries were carefully separated from the surrounding connective tissue. Then, after wrapping the carotid artery on both sides with a micro-coil (Sawane Spring Co., Hamamatsu, Japan) with an inner diameter of 0.18 mm, the skin was sutured with a suture line, and the area around the suture was disinfected with povidone. The approximate location where the micro-coil wraps around the carotid artery is depicted in Supplementary Figure S1. After the operation, when it was confirmed that they had sufficiently recovered from anesthesia, the mice were transferred to a recovery cage and then reared in the animal breeding room.

### 2.6 CRex oral gavage

The mice were randomly distributed into five groups to investigate the efficacy of CRex in a vascular dementia mice model. The experiment was performed by assigning seven mice to each experimental group. After excluding the mice whose body weight was significantly different from the average of the same experimental group, the results obtained from five mice in each experimental group were analyzed. The experimental groups comprised the Sham group, BCAS control group, and three CRex administration groups (CRex-5, CRex-50, and CRex-500) with three different concentrations of CRex (5 mg/kg, 50 mg/kg, and 500 mg/kg, respectively). The extract was resuspended in tap water and orally administered five times per week (Figure 1A).

### 2.7 Measurement of body weight and physiological parameters

During the experimental period, the mice were weighed weekly. Following behavioral measurements, blood samples were collected under deep anesthesia by cardiac puncture. To obtain serum, the blood samples were centrifuged at 1,500 *× g* for 15 min at 4°C. By measuring the serum concentrations of electrolytes, such as sodium (Na^+^), potassium (K^+^), and chloride (Cl^−^), using an electrolyte analyzer (Dri-Chem 3500i, Fuji, Japan), potential electrolyte imbalances were monitored.

### 2.8 Behavioral disorder assessment using the Y-maze test and the novel object recognition test (NORT)

The Y-maze is primarily used to test discriminant learning, working memory, and reference memory in animals (Kraeuter et al., 2019). On days 51, 53, and 55, i.e., a week before euthanization and brain collection, the mice were exposed to Y-maze acclimation training. Adaptive training allowed the subject to explore the Y-maze for 10 min using only two of three arms of the Y-maze (Figure 1B). A full-scale Y-maze experiment was performed at week 9, 1 day before sacrifice for brain harvesting. The mice were placed in the Y-maze for a total of 10 min: in the first 2 min, the mice adapted to the maze by exploring the two of three arms, and then the mice spent 8 min freely exploring the complete Y-maze. A score was given for entering each of the three arms (A, B, and C) of the Y-maze, and then calculated as the percentage spontaneous alternation under the following conditions: spontaneous Y-maze arm alternation was determined when successive selections of a triplet set (e.g., C–A–B, B–C–A, and A–B–C) resulted in entry into three arms. Spontaneous alternation behavior was calculated using the following formula: percentage alternation = ([number of alternations]/[total number of arm entries – 2]) × 100. Working memory is considered to be improved when the spontaneous alternation rate increases and the animals attempt to explore new objects.

NORT is used to assess a rodent’s preference for novel objects over familiar objects (Tahamtan et al., 2013; Denninger et al., 2018). During the adaptation phase, each mouse was allowed to explore the open field arena (40 × 40 × 40 cm (height) gray box) for 5 min without objects (Figure 1B). In the first trial, two identical objects (red circles in Figure 1B) were placed in the two opposite corners of the test arena and the animal was allowed to explore two objects for 10 min. After 20 min, in a second trial, the mice were placed in the arena again and one of the identical objects (familiar (F)) provided in the first trial was replaced with a novel (N) object. The behavior of each mouse was recorded for the 10-min period spent exploring each object. The analyses of object search time and discrimination rate (DR) were performed for each experimental group using the formula Total N h/(N h + F h) × 100. The two identical objects and arenas were washed with 70% ethanol between trials.

### 2.9 Euthanization, cardiac perfusion for brain harvesting, and cryosection of mice brain

The abdomen of the mice was excised and cardiac perfusion was performed with PBS. Briefly, the pulmonary artery was blocked, a 21-gauge needle was inserted into the left ventricle, and the needle was secured to the ascending aorta. Immediately after the perfusion was started, the right atrium was cut with scissors. PBS and 4% paraformaldehyde (PFA) were used for perfusion and fixation. For cryoprotection, the brains were sequentially immersed in 10%, 20%, and 30% sucrose solutions and stored in at −80°C frozen in OCT compound. Brain sections with a thickness of 25 μm were then obtained using a cryostat (Leica, Wetzlar, Germany). Sections were placed on glass slides for 12 h and stored at −80°C until use.

### 2.10 Immunofluorescence staining

The sections were dried on a slide warmer and incubated with blocking buffer (5% BSA) for 1 h at 25°C, washed with blocking buffer. Diluted primary antibodies against neuronal nuclear (NeuN), glial fibrillary acidic protein (GFAP), the cluster of differentiation 68 positive (CD68^+^) protein (Cat. nos. 94403, 12389, and 97778, respectively (Cell Signaling Technology, MA, USA), and tumor necrosis factor-alpha (TNF-α) (ab1793, Abcam, Cambridge, UK) were incubated with the sections overnight at 4°C. The primary antibodies were washed three times with PBS for 5 min, and the diluted secondary antibodies (goat anti-mouse or goat anti-rabbit IgG H&L, Abcam, Cambridge, UK) were added dropwise to the slides at 25°C for 2 h. The secondary antibodies were washed three times with PBS (5 min each wash). After adding the mounting medium dropwise with DAPI (ab104139, Abcam, Cambridge, UK), the sections were covered with cover slides, and the edges were sealed with nail polish. After observation using a fluorescence microscope (Ni-U, Nikon, Tokyo, Japan), the samples were stored in a refrigerator at 4°C for long-term storage. The positive cell count of immuno-intensity per unit area (1 mm^2^) of the brain was used for calculating marker expression. The fluorescence intensity observed after staining with an antibody was regarded as the protein expression level and was calculated using Digimizer version 4.6.1 (MedCalc Software Ltd, Ostend, Belgium).

### 2.11 Western blotting analysis

Proteins of the hippocampus and white matter parts of the brain were isolated using protein extracts. Lysates were obtained by centrifugation at 15,871 *× g* for 10 min at 4°C. The protein content of the supernatant was quantified using a BSA method. Proteins (30 μg) were resolved, separated by SDS-PAGE, and transferred to PVDF membranes (Millipore, Darmstadt, Germany). The membrane was blocked with 5% skimmed milk in TBST buffer for 1 h at 25°C and the expression of *p*-ERK, ERK, *p*-JNK, JNK, *p*-p38, p38, Wnt3a, *p*-GSK3β, GSK3β, and β-actin was detected. After overnight incubation, HRP-conjugated goat anti-rabbit IgG, pAb, and HRP-conjugated goat anti-mouse IgG pAb secondary antibodies were added for 1 h at 25°C. After incubation, the membrane was treated with a chemiluminescent solution and protein expression was detected using a light-sensitive luminescence spectrometry system (Amersham™ Imager 600, Amersham™, IL, USA). All bands were analyzed using the ImageJ program (NIH, MD, USA). The values were expressed as the ratio of the respective densities of phosphorylated and total protein bands.

### 2.12 RNA sequencing analysis

Total RNA was isolated using TRIzol reagent (15596058, InvitrogenTM, MA, USA) and RNA purity and ratios were assessed to confirm suitability for this study. We extracted mRNA from total RNA, synthesized cDNA, and performed mass sequencing. The expression level in the Sham group was set to 1, and the RNA expression level in the experimental samples was obtained by comparing the expression level of the BCAS control group and the BCAS combined with CRex-500 group with the expression level of the Sham group. Comparisons of relative expression levels were visualized as heat maps using the MeV program (https://mev.tm4.org). Additionally, after selecting the proteins for analysis, the STRING database (https://string-db.org) was used to construct protein–protein interaction (PPI) networks. Visualization was performed using Cytoscape (version 3.8.2), a software tool used for network analysis.

### 2.13 Statistical analysis

The experimental results of this study were analyzed using Sigmaplot 12.0 (Systat Software Inc.). The statistical significance of the difference in the means of each group was analyzed by one-way analysis of variance (ANOVA), with Dunnett’s method was adopted as a *post hoc* test method. As a result of the analysis, only cases with a *p*-value of less than 5% were judged to be statistically significant. All data in the experimental results were expressed as the mean ± standard deviation (SD).

## 3 Results

### 3.1 Network analysis for CR

**FIGURE 2:**
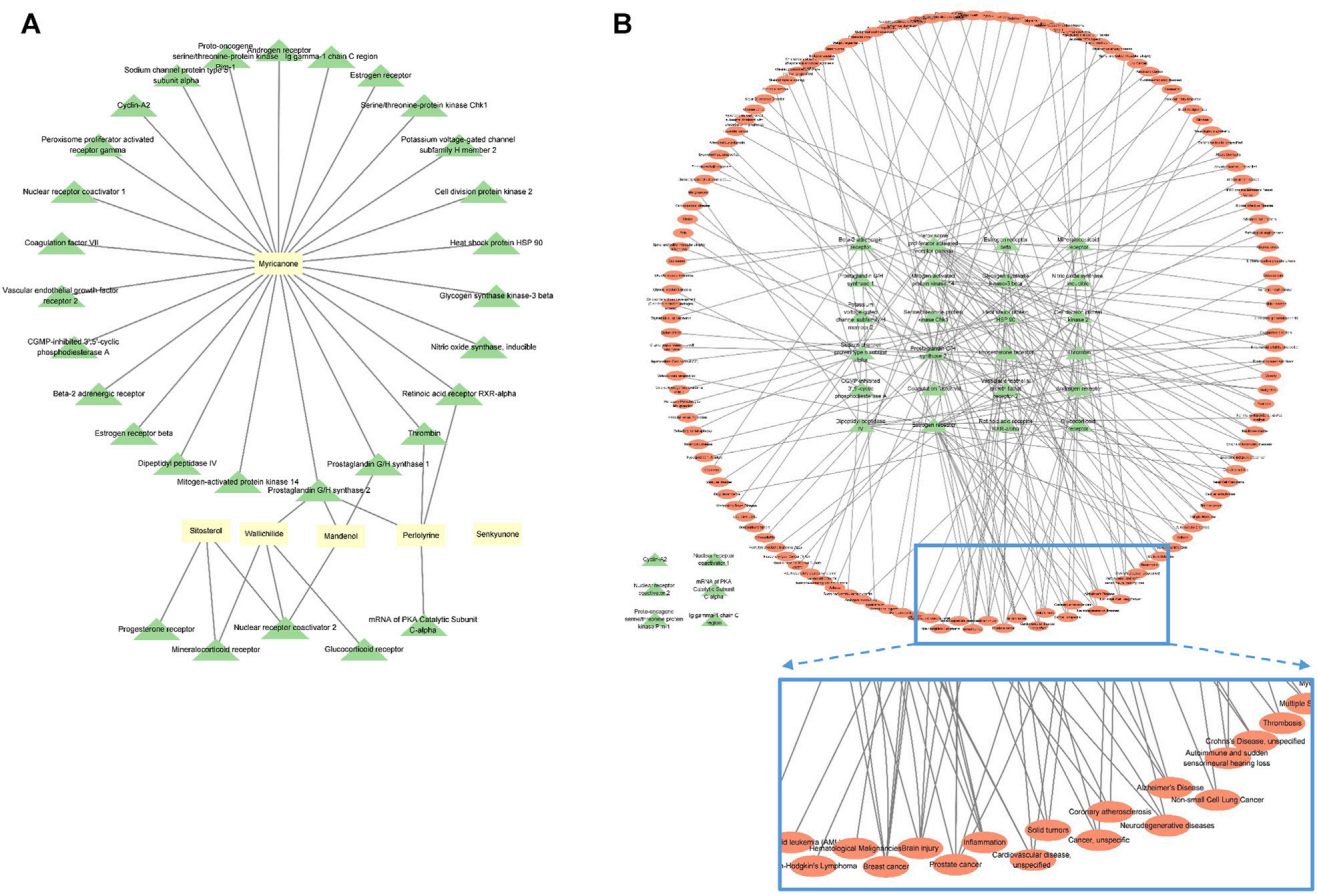
Network analysis of CR, revealing the compound–target protein network **(A)** and target protein–disease network **(B)**. Yellow rectangles represent compounds, green triangles represent target proteins, and red ovals represent diseases. The nodes inside the enlarged rectangle represent the diseases that have maximum connections with the target proteins.

For the components related to CR, 189 compounds were identified in the TCMSP database. After the selection of components using the AMDE variables, six compounds were extracted. Among these compounds, no target protein was identified for senkyunone (Supplementary Table S1; Figure 2A), and of the remaining five compounds, myricanone was found to have the most targets (Figure 2A). In total, thirty target proteins were expected to be regulated by the five compounds found (Figure 2A), and six of these proteins were not expected to be involved in diseases (Figure 2B). In total, 133 diseases were related to the remaining 24 proteins, and it was confirmed that brain, heart, and inflammation-related diseases were ranked highly among diseases with a large network of targets, whereas breast cancer and prostate cancer were not involved (Figure 2B). Through this analysis, it can be seen that CR can be effectively used for ischemic brain diseases such as cerebral infarction or VaD.

### 3.2 Changes in body weight and blood electrolytes

There was no difference in body weight changes among the experimental groups throughout the whole experimental period, which indicated that the surgery and drug administration to induce VaD did not cause significant physical and psychological stress to the experimental animals (Supplementary Figure S2A). The serum levels of electrolytes, such as sodium, potassium, and chloride, were not affected in the experimental groups. These results show that the surgical procedure and drug administration process would not impact the interpretation of the study results (Supplementary Figure S2B).

### 3.3 Measurement of behavioral changes

**FIGURE 3:**
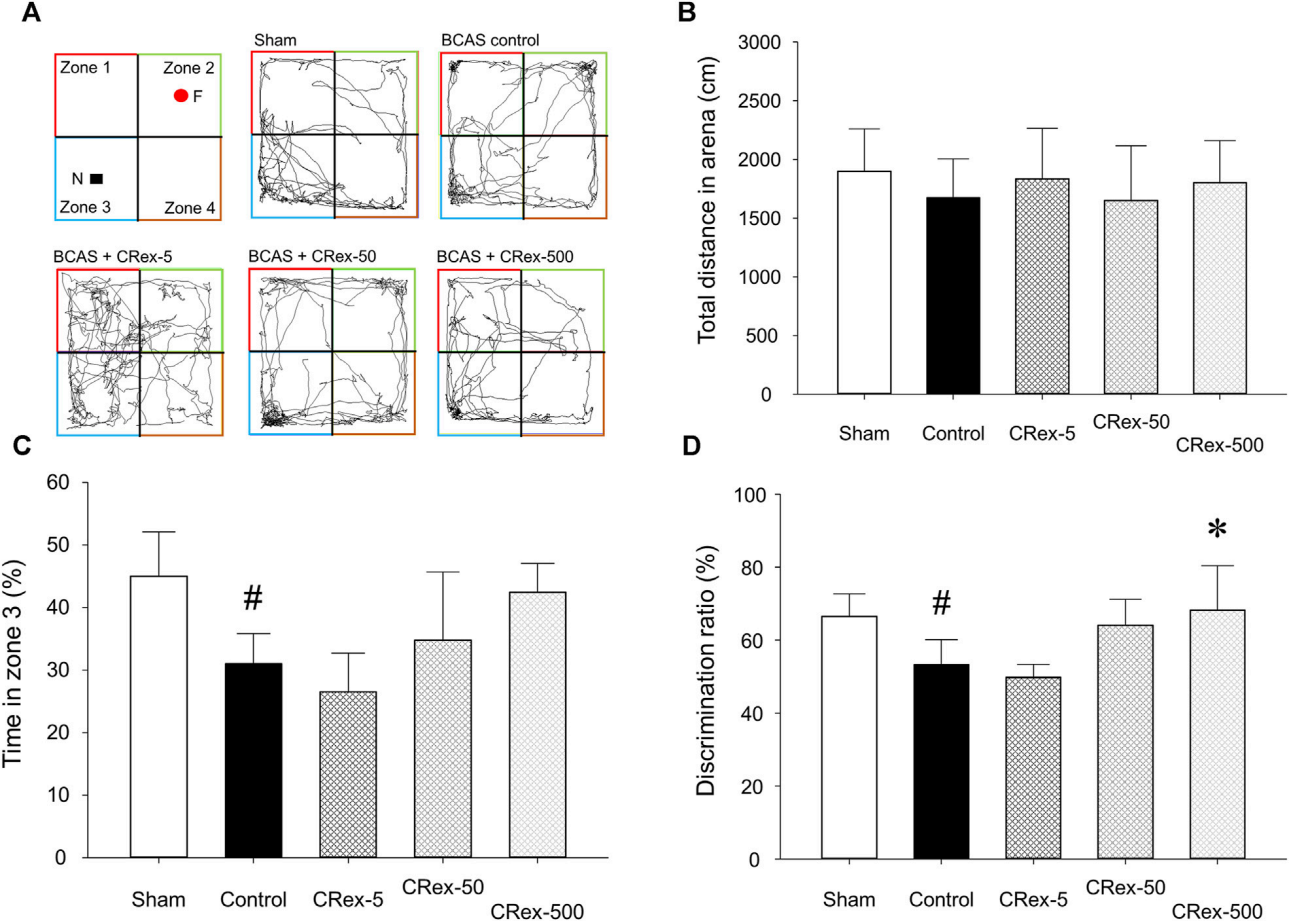
The path, total distance, percentage of time spent by experimental animals in zone 3, and percentage of time spent in zones 2 and 3 moving in the open field box. The path **(A)**, distance **(B)**, time spent in zone 3 **(C)**, and percentage of time spent in zones 2 and 3 **(D)**, where mice were exploring a novel object when the same object was placed in zones 2 and 3 and then changed to a novel object in zone 3, were analyzed. The results are presented as mean ± SD. ^#^ *p* < 0.05 vs. Sham group, *p < 0.05 vs. BCAS control group; *n* = 5 in each group.

When allowed to freely explore an open field box containing a novel object for 10 min, the total length of the path that each experimental animal moved did not differ among the experimental groups. However, in the BCAS control group, which underwent the BCAS operation, the exploration time in zone 3, where the novel object was located, was significantly decreased compared with the Sham group, and in the 500 mg/kg CRex treatment group, it was higher than in the BCAS control group, but the difference was not statistically significant (Figures 3A–C). When comparing the ratio of time spent in zone 2, with a familiar object, and zone 3, with a novel object, it was significantly lower in the BCAS control group compared with the Sham group, but significantly higher in the CRex-500 group (Figure 3D). In the Y-maze test, there was no difference in spatial recognition ability among the experimental group; notably, in the BCAS control group, this ability was lower compared with the Sham group, but the difference was not statistically significant (Supplementary Figure S3). This was because the standard deviation was relatively large, which appeared to be due to the difference in spatial perception ability among experimental animals.

### 3.4 Expression changes in microglia and astrocytes in the hippocampus and white matter of mouse brain

**FIGURE 4:**
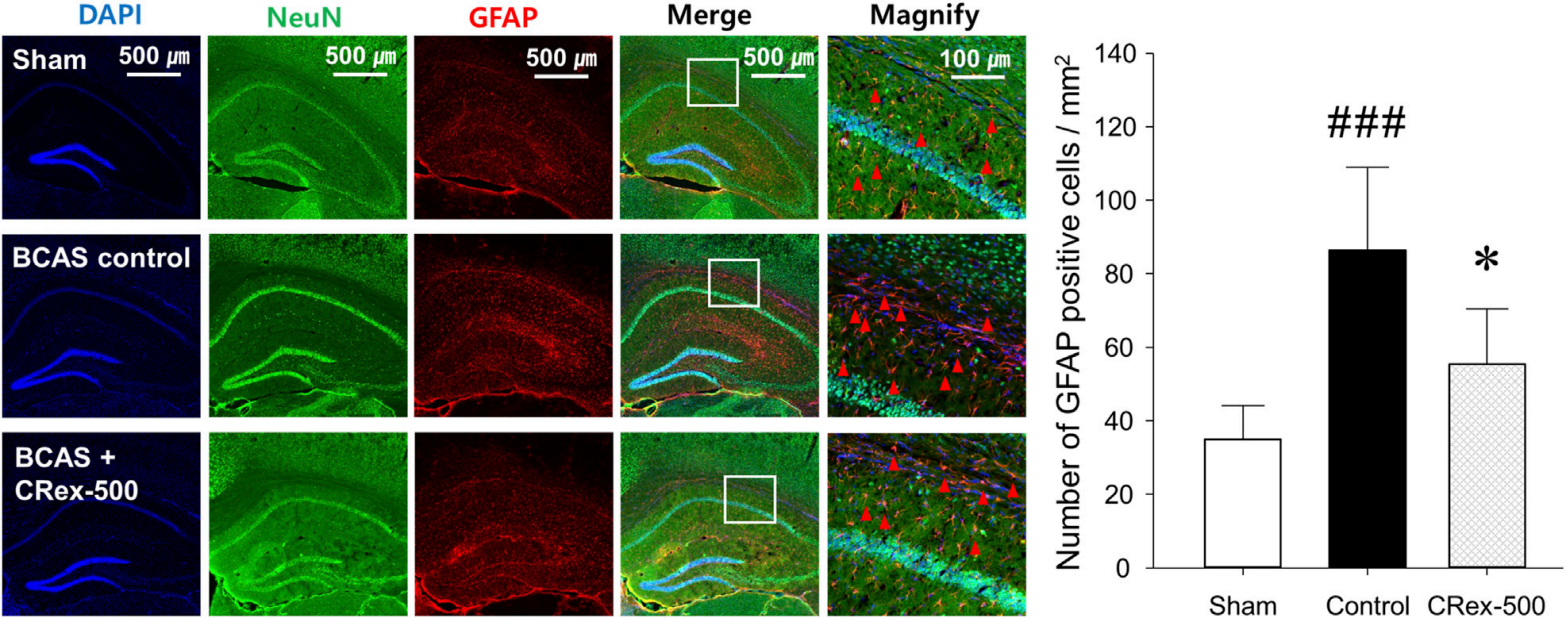
Observation of astrocyte activation. To confirm the expression of GFAP (red arrow heads), a marker of astrocytes, NeuN, and DAPI were counterstained to observe the distribution of astrocytes. The right hippocampus and corpus callosum were observed at ×40 and ×200 magnification, and the changes in the corpus callosum area were confirmed through the magnified images. The results are presented as mean ± SD. ^###^ *p* < 0.001 vs. Sham group, *p < 0.05 vs. BCAS control group; n = 5 in each group.

**FIGURE 5:**
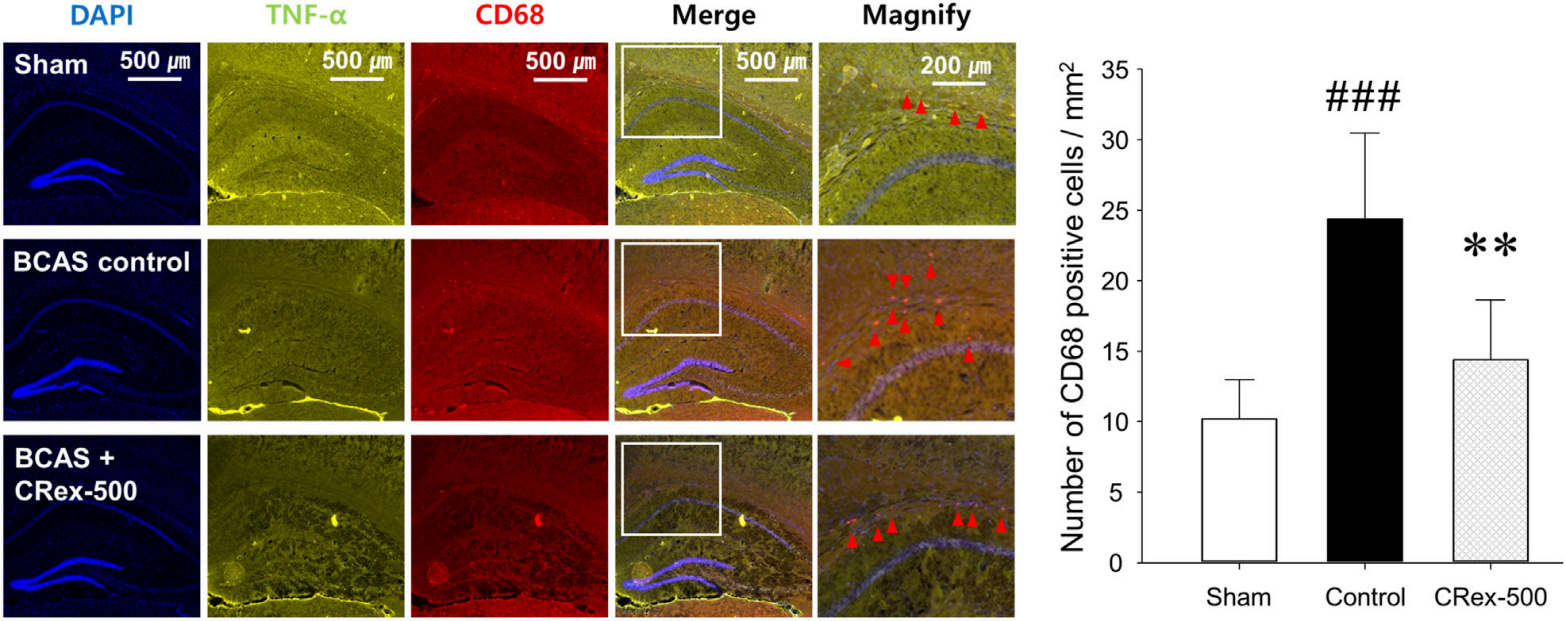
Observation of microglial activation. To confirm the expression of CD68 (red arrow heads), a marker of microglia, DAPI was counterstained to observe the distribution of astrocytes, and TNF-α expression was confirmed. The right hippocampus and corpus callosum were observed at ×40 and ×100 magnification, and the changes in the corpus callosum area were confirmed through the magnified images. The results are presented as mean ± SD. ^###^ *p* < 0.001 vs. Sham group, **p < 0.01 vs. BCAS control group; *n* = 5 in each group.

To confirm the expression of astrocytes and microglia, which are the most representative cells involved in inflammatory response in the brain, immunofluorescence staining was performed. As shown in Figure 4, the distribution of neurons was evenly observed in the Sham group, but the degree of staining of neurons was weak around the white matter in the BCAS control group. Interestingly, it was also found that GFAP, a representative marker of astrocytes, was strongly expressed in the BCAS control group, and the staining of pyramidal cells in the hippocampus adjacent to this area was relatively weak. In contrast, when a relatively high concentration of CRex was administered (CRex-500 group), the expression of GFAP decreased and the staining of neurons was relatively uniform. CD68, a marker of the microglia, also appeared to be more intensely stained in the BCAS control group than in the Sham group, and it was found that this expression decreased in the CRex administration groups (Figure 5).

### 3.5 Western blotting analysis

Mitogen-activated protein kinase (MAPK) is a mechanism that plays an important role in the processes of cell proliferation, differentiation, and apoptosis, and the major proteins that constitute this mechanism are ERK, JNK, and p38 (Doust et al., 2021; Faraji et al., 2009; Jiwa et al., 2010; Lee et al., 2019; Liu et al., 2021; Nakaji et al., 2006; Nishio et al., 2010; Ono et al., 2010; Sawe et al., 2008; Sun and Nan, 2016; Sun, Y. et al., 2015; Vorhees and Williams, 2014; Widmann et al., 1999). In this study, we used Western blotting to examine how MAPK signaling affects the occurrence of VaD, the changes in cognitive function caused by BCAS, and whether the administration of CRex could reduce the pathological changes. Consequently, we confirmed that the phosphorylation of ERK and p38 proteins was increased significantly by BCAS, and that 5 mg/kg CRex administration inhibited the phosphorylation of ERK, whereas 500 mg/kg CRex administration inhibited phosphorylation of the p38 protein. In this study, BCAS caused no change in the phosphorylation of JNK; interestingly, the JNK phosphorylation was decreased by 5 mg/kg and 500 mg/kg CRex administration.

According to the many studies on AD and a few studies on VaD, there are many cases in which it is reported that the inhibitory phosphorylation of the GSK3β protein is decreased and the canonical Wnt signaling or the expression of Wnt3a is decreased in animal models; many published studies show that the regulation of these two proteins is necessary (Zhang et al., 2016; Guo et al., 2019; Kumar et al., 2019; Zhao et al., 2019; Sayas and Ávila, 2021; Wang et al., 2021; Chauhan et al., 2022; Kumari et al., 2022; Yang et al., 2022). However, in this study, the expression levels of Wnt3a protein and the inhibitory phosphorylation of GSK3β protein in mice are increased by BCAS (Figure 7), which was different from the previous studies. The administration of CRex inhibited the changes in protein expression caused by BCAS. Therefore, more detailed studies of the expression of GSK3β and Wnt3a under various conditions in animal models of VaD are needed.

### 3.6 Analysis of gene expression and protein interaction using RNA sequencing

To confirm whether CRex can regulate the changes in pathological gene expression in the hippocampus and the white matter periphery of mice subjected to BCAS, we used RNA sequencing to examine the overall trend in genes with changed expression. In total, 23,282 genes were targeted, and genes with two-fold changes in pathological expression were selected. The clustering of genes with pathological changes in expression is presented in Supplementary Figure S4 to allow an understanding of their expression patterns. The gene expression level in the Sham group was set as 1; red genes were increased more than two-fold in expression in the experimental groups, and green genes were decreased to less than half of the expression of the sham group. Genes marked in black after the administration of 500 mg/kg CRex were genes that suppressed pathological changes, that is, genes that were not significantly different from the Sham group. For the quantitative analysis of these changes, we plotted a graph of the changes in expression of the individual genes. Following BCAS, the number of pathological genes that increased was 1,174, and the number of pathological genes that decreased was 745. Of the 1,919 genes that showed pathological changes, 847 were regulated by CRex, which means that CRex administration regulated approximately 44% of genes to normal levels (Figures 8A–C). We searched for proteins involved in these genes regulated by CRex administration and constructed a protein interaction network of these proteins. We used the STRING database for analysis and separately constructed networks between proteins related to the downregulated genes and proteins related to the upregulated genes, and checked which proteins interacted with many other proteins. The results showed that sonic hedgehog (Shh), lymphocyte-specific protein tyrosine kinase (Lck), transition protein 1 (Tnp1), etc., were proteins that interacted a lot in the analysis of downregulated genes (Figure 9A), and toll-like receptor 2 (Tlr2), Kit (KIT proto-oncogene), RAD51 recombinase (Rad51), etc., were proteins that interacted a lot in the analysis of upregulated genes (Figure 9B).

## 4 Discussion

The present study aimed at investigating whether CRex can suppress the symptoms of VaD in mice and present supporting result analysis. First, on referring to existing studies on CR, we found the evidence that CR can be used for treating VaD from the TCMSP database. Subsequently, we induced VaD using BCAS model in mice and administered CRex. The CRex was found to be effective in treating VaD in mice from the improvement in cognitive dysfunction caused by VaD.

VaD refers to dementia caused by damage to brain tissue due to CVD. CVD can be divided into ischemic CVD (cerebral infarction or cerebral ischemia) and hemorrhagic CVD (cerebral hemorrhage) depending on the mechanism of occurrence (Biessels and Despa, 2018; Craft, 2009; O'Brien and Thomas, 2015). As the number and proportion of elderly people in society gradually increase owing to aging, the number of dementia patients increases and the duration of illness also increases, which not only increases the burden of care for dementia patients, but also imposes a heavy burden on medical insurance finances. The measures for dementia prevention and treatment are always important issues of interest because of the increasing mental, physical, and economic burden on the patients suffering from this disease and the families who support these patients (Allan et al., 2011; Connors et al., 2020). It is known that AD accounts for a large proportion of dementia in the West, whereas the proportion of VaD is much higher in the East than in the West (Kalaria et al., 2008; Wolters and Ikram, 2019); moreover, it has been reported that the burden of care for families of patients with VaD is much higher than for families of patients AD in early stage of each type of dementia (Vetter et al., 1999). This is because physical disabilities impacting daily life often occur with VaD.

The plant of origin of CR, *C. anthriscoides* “Chuanxiong,” is a perennial herbaceous plant belonging to the Umbelliferae family. It is widely cultivated in China, Korea, and Japan, and the young leaves of this plant are eaten as vegetables and the rhizomes and roots are known to expand blood vessels and activate blood circulation in traditional Asian medicine (Gao et al., 2017; Su et al., 2021; Yang et al., 2020). Recent results have shown that CR or its components have various activities, such as anticancer, analgesic, anticonvulsant, anti-inflammatory, sedative, vasodilatory activities (Li et al., 2012; Du et al., 2016; Wang et al., 2022). Recently, Wang et al. (2020) reported the induction of cerebral infarction in mice and showed the effects of the administration of CR ethanol extract. They elucidated the mechanism by which CR extract inhibits the inflammatory response caused by ischemia and inhibits apoptosis. Zhao et al. (2018) used CR for a VaD preclinical study, inducing VaD in rats by ligating both carotid arteries and administering a single component extracted from CR, ligustrazine, and reported that it inhibited the decrease of neurotrophic factor in the hippocampus area of the brain by regulating the proteins involved in apoptosis.

In this study, we analyzed whether CR could be effectively used for VaD through network analysis; then, we selected six components, including myricanone; identified proteins that could be regulated by these components; and confirmed the diseases associated with changes in these proteins. As indicated above, CR is often used to treat CVDs in Asian medicine; however, little research has been conducted on its potential use dementia treatment, including VaD. Since herbal medicines contain several metabolites, they can be used for variety of diseases. Particularly, to determine whether CR can be used for treating cerebral diseases such as VaD, we identified compounds that are expected to be active and identified the target proteins and diseases on which these compounds act. The results indicated that CR could be effective in inflammatory diseases occurring in the brain (Figure 2).

The studies using existing activity research databases may show different results in actual animal models, thereby necessitating the direct confirmation of the effects using an appropriate animal model. Therefore, to confirm whether it was effective in an animal model of VaD, we performed a preliminary study using various VaD models in mice and found that the method of reducing carotid artery to a certain diameter using micro-coil was the most reproducible; thus, we selected this method. When a micro-coil with an inner diameter of 0.18 mm was wound around both carotid arteries of mice, a chronic reduction in blood supply to the brain due to narrowing of carotid arteries causes pathological changes resulting from decreased cerebral blood flow (CBF) and leading to long-term cognitive impairment (Lee et al., 2019; Liu et al., 2021; Selvaraji et al., 2022; Ishikawa et al., 2023). After BCAS surgery, no treatment was performed for 2 weeks to allow the pathology arising from decreased CBF to manifest, and then three concentrations (5, 50, and 500 mg/kg, respectively) of CRex were administered five times per week for 6 weeks, from the third experimental week to the eighth week, and the results were observed. We tried to examine changes in short-term spatial working memory owing to VaD using the Y-maze test, but there was no significant difference between all experimental groups in this study. The Y-maze test is a method that can measure spatial working and reference memory, and many studies have been conducted using this method (Hu et al., 2012a; Hu et al., 2012b; Kraeuter et al., 2019), but there was no statistically significant difference identified in this study because when statistical processing was performed for individual test results, the SD was too large (Supplementary Figure S3). However, significant differences were observed in NORT in the BCAS control group compared with the Sham group (Figure 3). NORT is one of the research methods that is used to analyze recognition memory in rodent models of CVD by measuring the time spent exploring novel objects and familiar (Tahamtan et al., 2013; Denninger et al., 2018; Choi et al., 2022). There was no difference in the total distance traveled in each experimental group (Figure 3B), but when comparing time spent exploring familiar objects in zone 2 and novel objects in zone 3, the exploration time was significantly lower in the BCAS control group compared with the Sham group, and this decrease was suppressed in the experimental group administered 500 mg/kg CRex (Figure 3D). Therefore, it was confirmed that CRex has the ability to reduce cognitive impairment caused by chronic CBF reduction.

**FIGURE 6:**
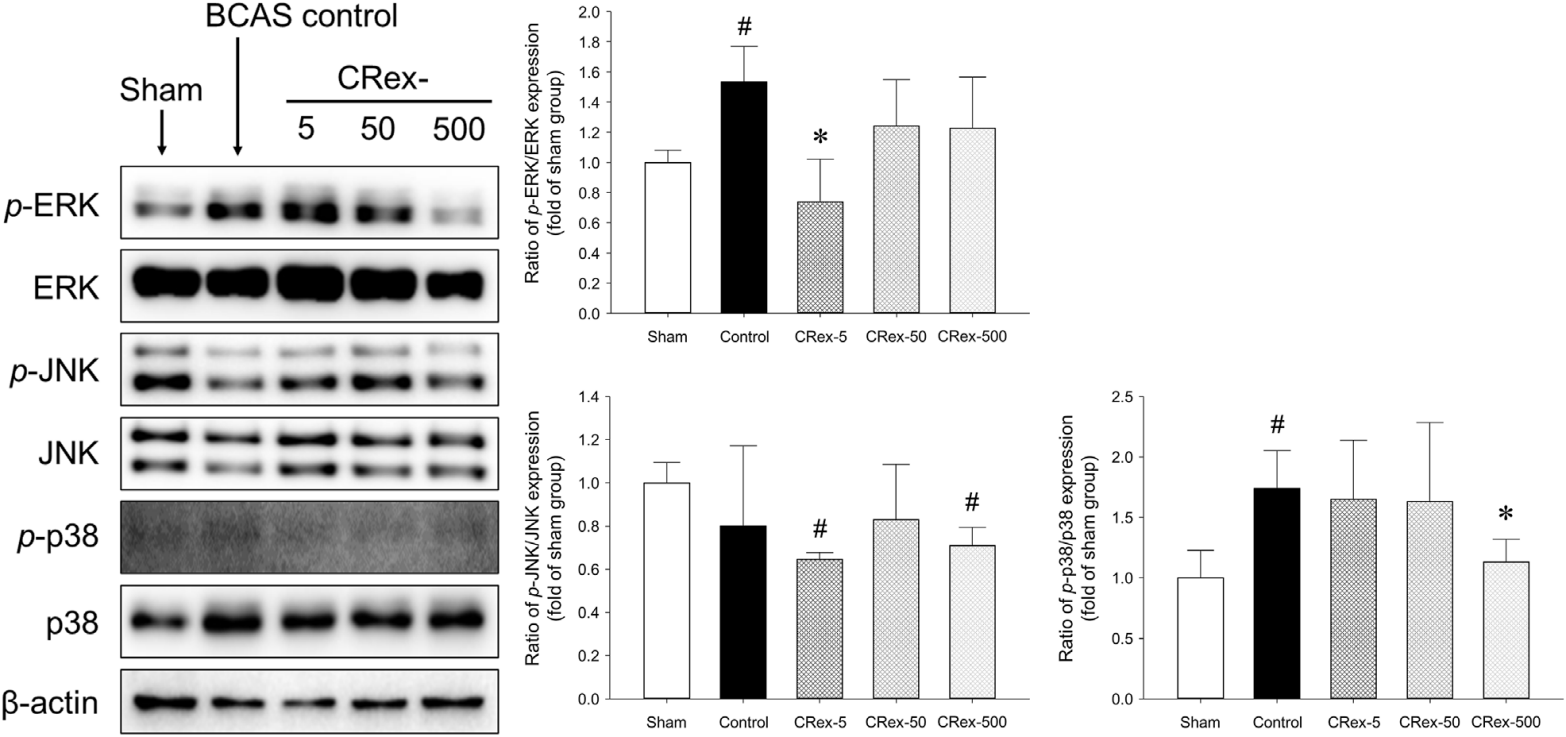
Expression of proteins involved in MAPK pathway. The expressions of the major proteins, MAPK, ERK, JNK, and p38, were quantified, and relative expression of the phosphorylated protein to the total protein was used to indicate the degree of expression. The results are presented as mean ± SD. ^#^ *p* < 0.05 vs. Sham group, *p < 0.05 vs. BCAS control group; n = 5 in each group.

**FIGURE 7:**
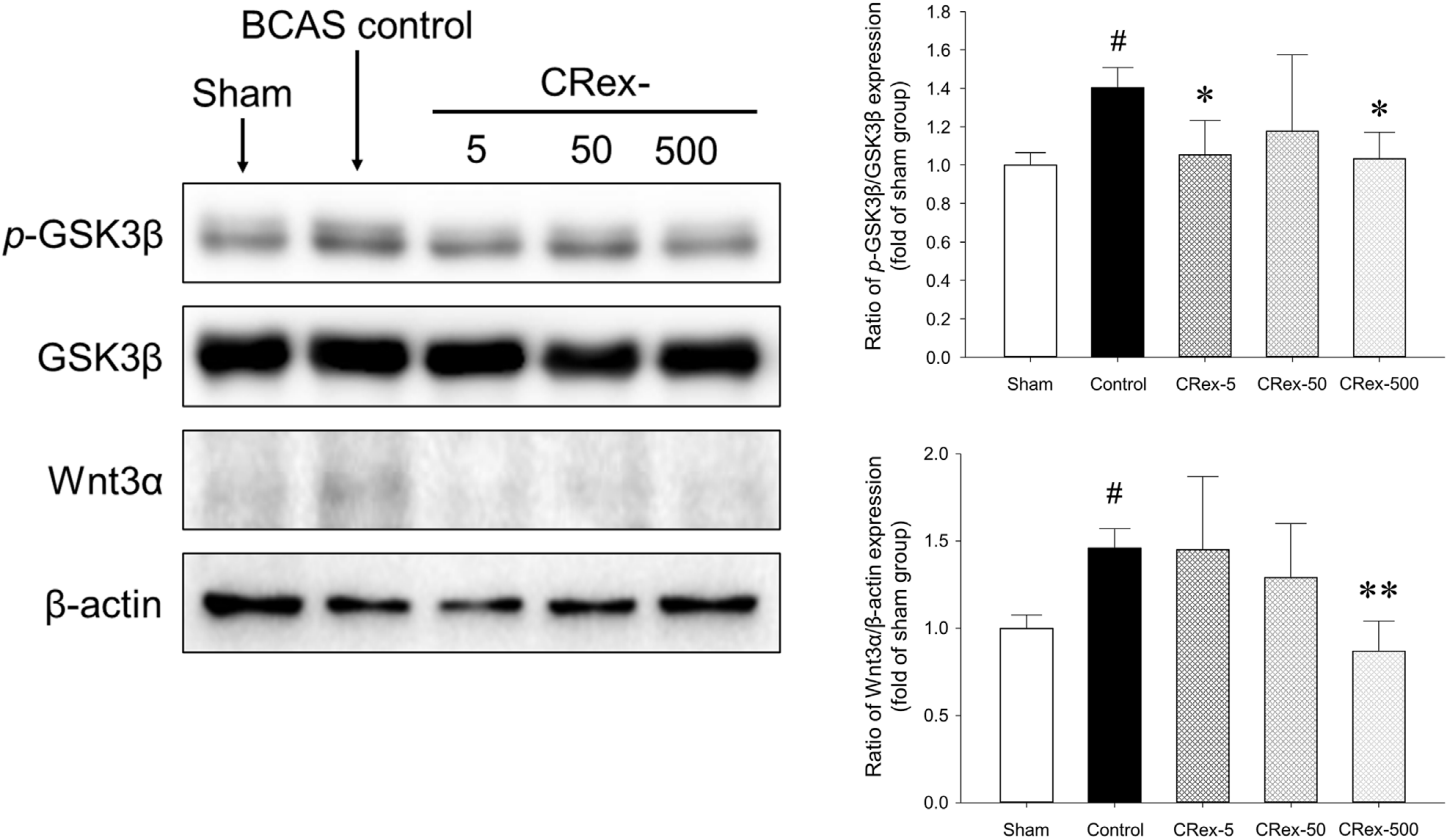
Measurement of the expression of GSK3β and Wnt3a proteins. The expression of the GSK3β protein was quantified and the expression of phosphorylated GSK3β protein relative to the total protein was compared. Additionally, the expression of Wnt3a protein was compared with amount of β-actin. ^#^ *p* < 0.05 vs. Sham group, *p < 0.05 vs. BCAS control group, **p < 0.01 vs. BCAS control group; *n* = 5 in each group.

The hippocampus, which is known to be responsible for learning and memory, is located inside the temporal lobe of the brain and is known to work on regulating learning, memory encoding, memory consolidation, mood regulation, spatial navigation, etc., and is a critical aspect of dementia research (Faraji et al., 2009; Nishio et al., 2010; Ono et al., 2010; Vorhees and Williams, 2014; Doust et al., 2021). However, it was recently reported that white matter damage is one of the important factors involved in dementia (Nakaji et al., 2006; Faraji et al., 2009; Jiwa et al., 2010; Nishio et al., 2010; Ono et al., 2010; Vorhees and Williams, 2014; Lee et al., 2019; Doust et al., 2021; Liu et al., 2021). Therefore, in this study, we separated both the hippocampus and the white matter, the corpus callosum, and performed Western blotting to examine the changes in proteins involved in MAPK signaling. Consequently, the phosphorylation of ERK and p38 proteins increased significantly by BCAS, and CRex administration suppressed this increase. However, the phosphorylation of ERK was inhibited by 5 mg/kg CRex, and p38 was inhibited by 500 mg/kg CRex (Figure 6). In mice with white matter lesions, MAPK and ERK pathway expression was enhanced, which results in neuronal death (Guo Y et al., 2020), and numerous pathways such as p38 MAPK and ERK are involved in triggering astrocytes and microglia to secrete inflammatory cytokines in neurodegenerative diseases including AD (Rai et al., 2019; Thakur et al., 2023). This study confirmed that these signaling pathways were functional in VaD mice model as well, and administration of CRex inhibited the phosphorylation of above proteins. Thus, it is evident that CRex suppresses damage to cognitive function by reducing death of neuronal cells induced by BCAS. The inhibitory phosphorylation of GSK3β and Wnt protein downregulation are known to be involved in neuronal damage (Zhang et al., 2016; Jaworski et al., 2019; Qu et al., 2020; Sayas and Ávila, 2021; Wang et al., 2021; Kumari et al., 2022), but in this study, the expression levels of the proteins were higher in the Sham group by BCAS, and this increase was suppressed by CRex administration (Figure 7). In our study, only the hippocampus and white matter were used as subjects for Western blotting, but an explanation reason for the contradictory results with other researchers cannot be clearly explained. It is necessary to observe the expression level of proteins and genes in different parts of the brain through follow-up studies.

Studies on brain function are focused mainly on neurons, but research into non-neuronal cells such as astrocytes, oligodendrocytes, and microglia is also important to effectively interpret brain aging and inflammation. Many studies have reported that astrocytes activate microglia and cause neuroinflammation, leading to neuronal degeneration and cognitive impairment resulting from brain aging (Pekny and Nilsson, 2005; Hirayama et al., 2015; Hoogland et al., 2015; Lana et al., 2020; Doust et al., 2021). These results suggest that astrocytes and microglia are stable in normal conditions and protect neurons; however, depending on the situation, they change into reactive astrocytes with neurotoxicity and cause neuroinflammation reactions mediated by microglia, resulting in damage to the adjacent neurons. In this study, we observed brain tissue around the hippocampus in CRex-500 group that showed a clear improvement in cognitive function and could identify that the action occurred through MAPK signaling (Figures 3, 6). However, no pathological changes were observed in the CA1 area of the hippocampus, although neuronal loss and astrocyte activation were observed in the corpus callosum adjacent to this area (Figure 4). Additionally, the overexpression of microglia was confirmed (Figure 5). Astrocytes are mainly distributed in the gray matter and white matter in the brain. Several research results have demonstrated degenerative changes in the white matter when dementia occurs (Pekny and Nilsson, 2005; Hirayama et al., 2015; Lana et al., 2020). In this study, the over-expression of GFAP positive astrocytes and loss of NeuN positive neurons in the white matter and adjacent area were clearly observed in mice model of VaD by BCAS. Moreover, as CRex suppressed this expression, it is believed that it will be possible to develop a therapeutic agent against VaD. Conclusively, the damage to the corpus callosum area was confirmed to occur by BCAS in this study, and it appears that this damage was mainly involved in the neuroinflammation reactions of astrocytes and microglia.

**FIGURE 8:**
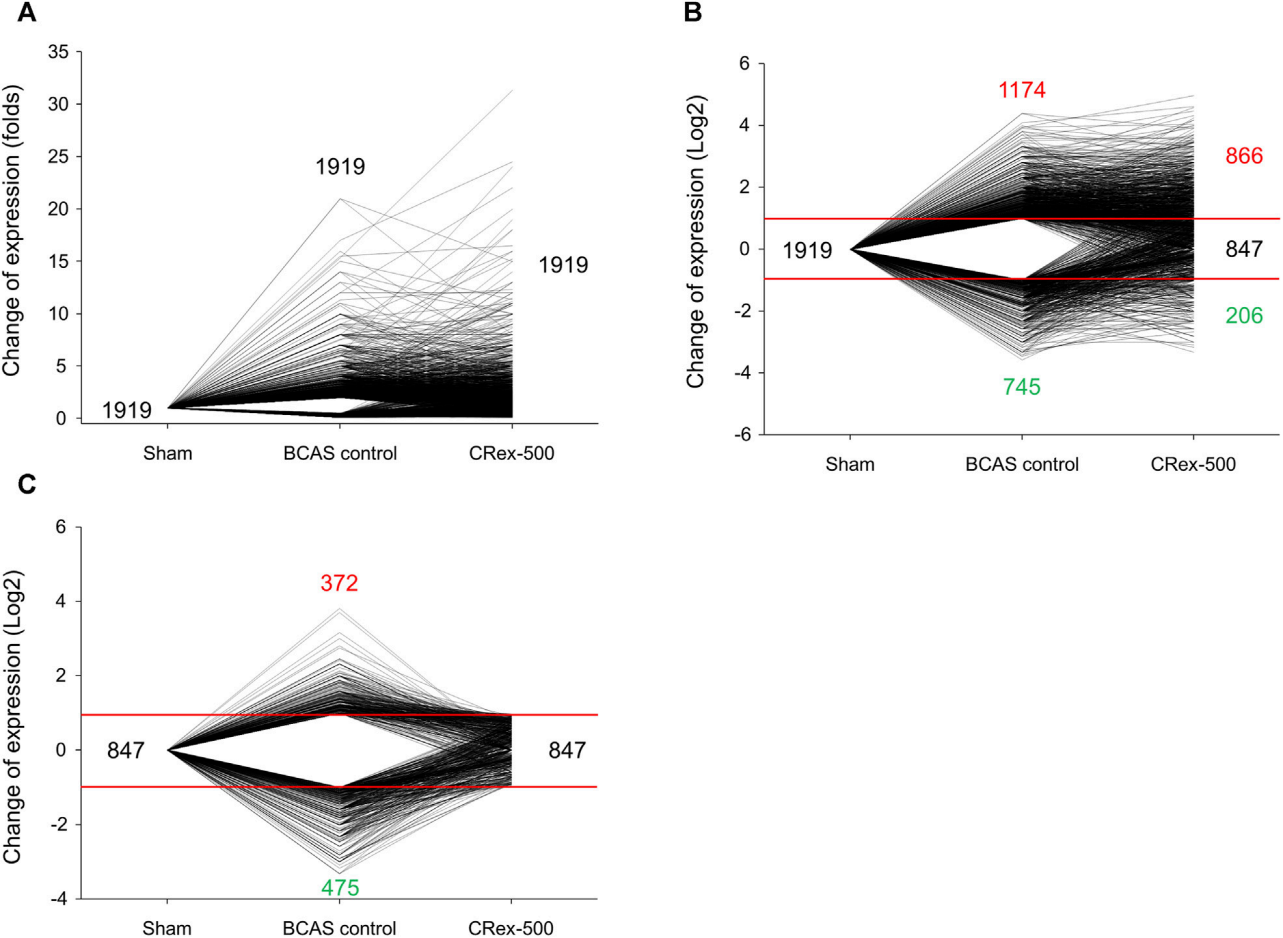
Analysis of trends in gene expression after BCAS or 500 mg/kg CRex administration. **(A)** The fold change in gene expression in experimental groups when the gene expression in the Sham group was considered as 1. **(B)** Log-transformed values, enabling the increases and decreases to be visualized. **(C)** The expression of genes regulated by CRex administration in each experimental group.

**FIGURE 9:**
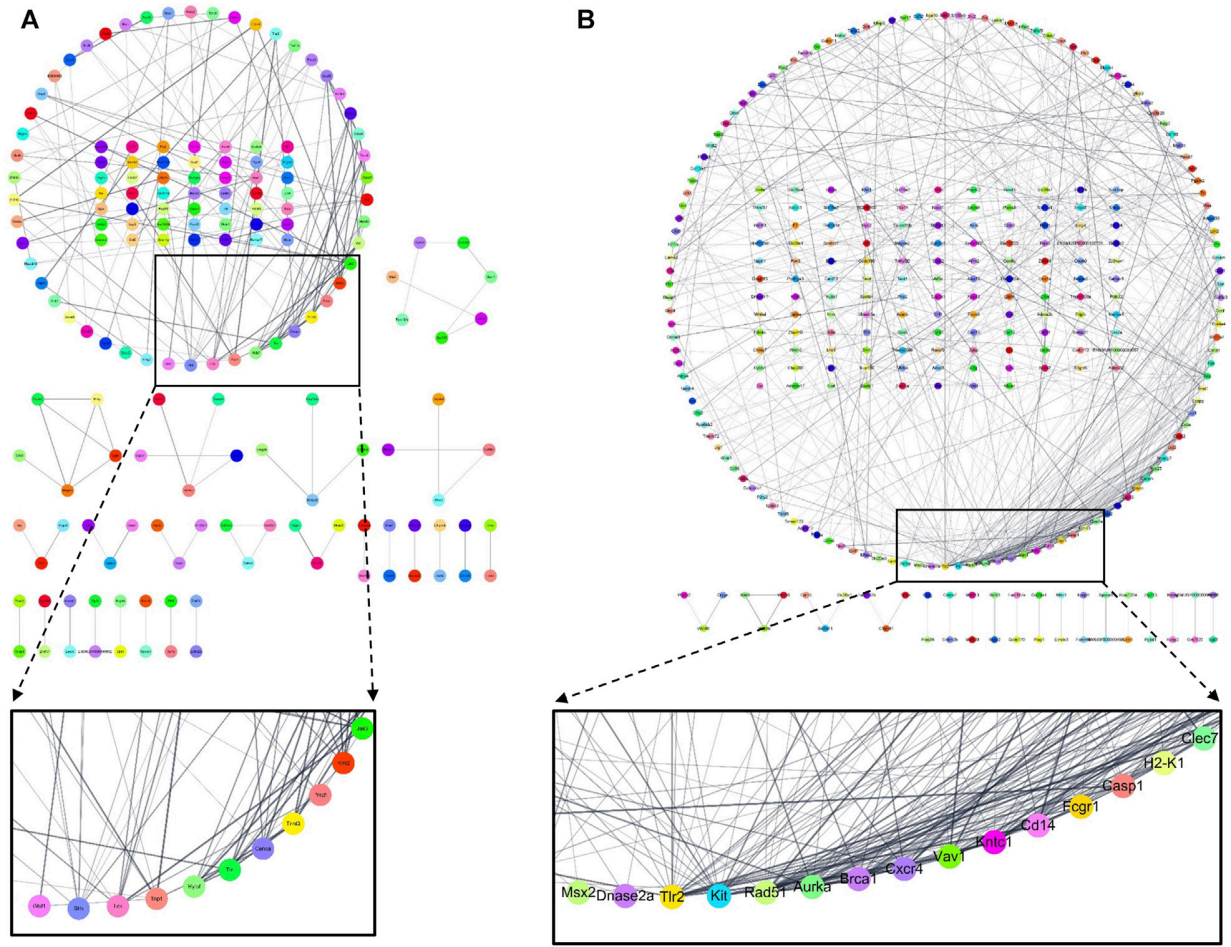
The network of protein–protein interaction (PPI) composed of related genes regulated by 500 mg/kg CRex administration. PPI networks related to genes that were increased **(A)** or decreased **(B)** by BCAS but were regulated by CRex administration have been presented. Using the STRING database, we derived the proteins that play a central role by constructing PPI networks related to genes altered in expression by CRex administration.

As herbal medicines are composed of numerous components, rather than exerting activity through a few mechanisms, there is a greater possibility that activity occurs through many pathways; therefore, in this study, we observed the expression of many genes at the same time to determine the pathological gene expression induced by BCAS and to see if CRex suppressed these changes, and to what extent it inhibits the pathological expression of genes. We performed RNA sequencing of samples from the hippocampus and corpus callosum of mice and confirmed the gene expression pattern (Supplementary Figure S4). Out of approximately 23,000 genes, 1,919 genes changed in expression by more than twice or less than half following BCAS, and 847 genes, which accounted for approximately 44% of these 1,919 genes, were regulated to within the normal range (Figure 8). Therefore, we could see that the expression of many genes was regulated by CRex, and we constructed an interaction network of proteins related to these genes and confirmed that proteins such as Shh, Lck, Tnp1, Tlr2, Kit, Rad51, etc., may play major roles in this network (Figures 9A, B). Shh is known to be involved in cell growth and shape formation and it has also been reported to be involved in neurogenesis in the adult hippocampus (Li, X. et al., 2021; Yao et al., 2016). Lck stabilizes Rad51 and acts to repair DNA damage (Dey et al., 2023), and Tlr2 is involved in the inflammatory response (Wang et al., 2013; Miyata, 2022), showing that various mechanisms occur simultaneously to induce the symptoms of BCAS and their subsequent amelioration following drug administration.

**FIGURE 10:**
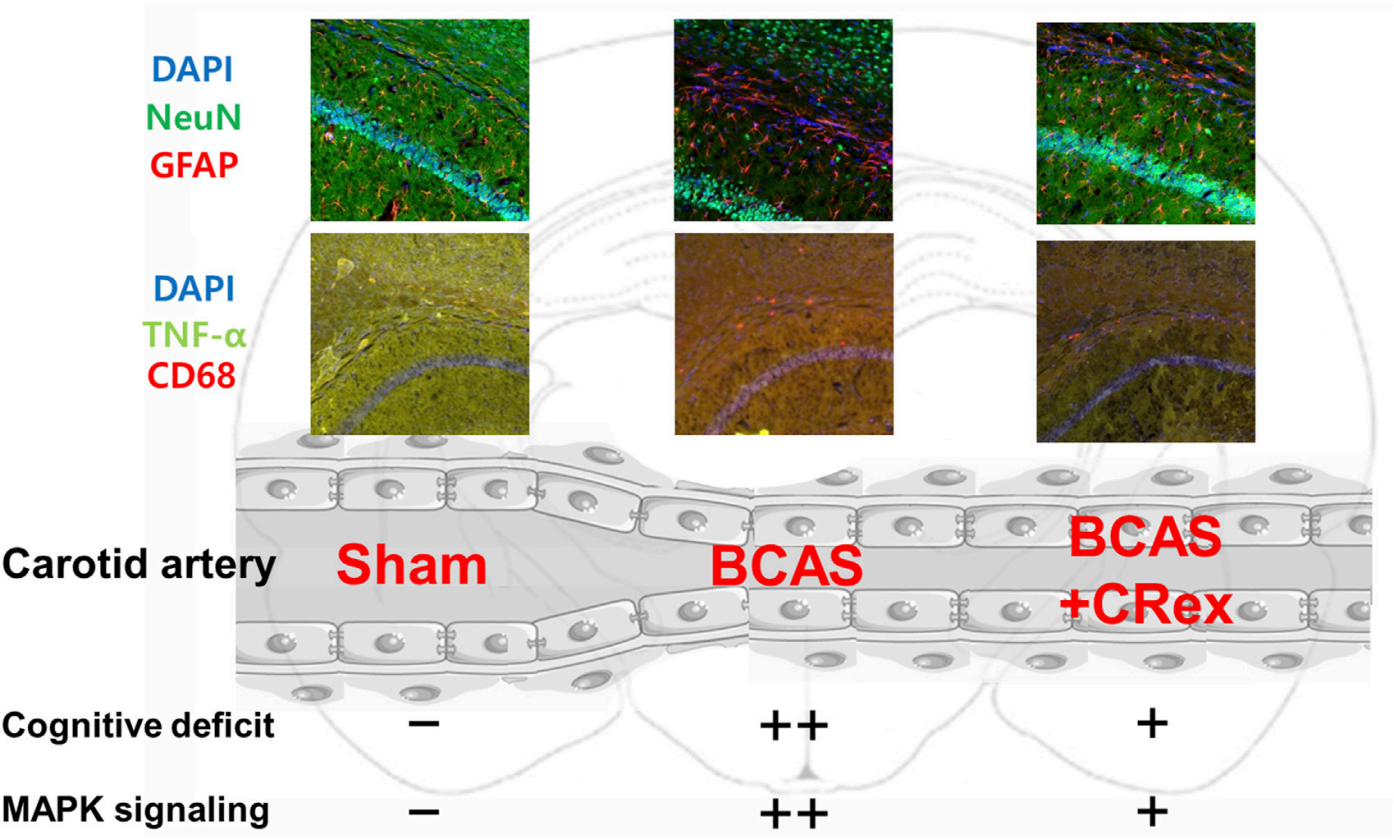
Graphical abstract. The activation of astrocytes and microglia was enhanced by BCAS, and this activation was suppressed by administration of CRex. It is presumed that the impairment of cognitive function was also prevented by inhibition of the activation of inflammatory cells by CRex. Additionally, the expression of MAPK, one of the primary proteins in diverse cellular mechanisms, was increased by BCAS, and CRex also reduced this expression.

In summary, CR, which has a long history of use as a traditional Asian medicine because of its effectiveness for CVD, was applied to treat VaD. CR suppressed the cognitive impairment caused by VaD and inhibited the damage to white matter around the hippocampus caused by the activation of astrocytes and microglia, and reduced pathological changes in many genes (Figure 10). Therefore, through this study, it was confirmed that CR is effective for the treatment of ischemic CVD, including VaD.

## Data Availability

The original contributions presented in the study are included in the article/Supplementary Material, further inquiries can be directed to the corresponding author. The data presented in the study are deposited in https://data.mendeley.com/preview/8792mk7d45?a=b32fd3f0-a89e-4483-9664-64b7eaf7c12d and the Science Data Bank (www.scidb.cn), accession number 31253.11.sciencedb.16109.

## Abbreviations

CVD, cerebrovascular disorders; VaD, vascular dementia; AD, Alzheimer’s disease; CR, Chuanxiong Rhizoma; CRex, CR methanol extract; BCAS, bilateral common carotid artery stenosis; TCMSP, Traditional Chinese Medicine Systems Pharmacology Database and Analysis Platform; NORT, novel object recognition test; IF, immunofluorescence; ANOVA, one-way analysis of variance; MAPK, mitogen-activated protein kinases.
