# Supplementary material for: Methanol extract of Ligusticum chuanxiong Hort. Rhizome ameliorates bilateral common carotid artery stenosis-induced cognitive deficit in mice by altering microglia and astrocyte activation
Source: Front Pharmacol. 2024 Mar 14;15:1329895. doi: 10.3389/fphar.2024.1329895 (PMC10973115; doi:10.3389/fphar.2024.1329895)
Supplement: Supplementary file 1 [file Table1.DOCX]

Table S1. List of potentially bioactive compounds in Chuanxiong Rhizoma (CR) identified from the TCMSP database.

| Mol ID | Molecule name | Molecular weight | OB (%) | Caco-2 | DL | BBB |
| --- | --- | --- | --- | --- | --- | --- |
| MOL000359 | [Sitosterol](https://tcmspw.com/molecule.php?qn=359) | 414.79 | 36.91 | 1.32 | 0.75 | 0.87 |
| MOL002135 | [Myricanone](https://tcmspw.com/molecule.php?qn=2135) | 356.45 | 40.60 | 0.67 | 0.51 | −0.08 |
| MOL001494 | [Mandenol](https://tcmspw.com/molecule.php?qn=1494) | 308.56 | 42.00 | 1.46 | 0.19 | 1.14 |
| MOL002157 | [Wallichilide](https://tcmspw.com/molecule.php?qn=2157) | 412.57 | 42.31 | 0.82 | 0.71 | 0.73 |
| MOL002140 | [Perlolyrine](https://tcmspw.com/molecule.php?qn=2140) | 264.3 | 65.95 | 0.88 | 0.27 | 0.15 |
