# Supplementary material for: Methanol extract of Ligusticum chuanxiong Hort. Rhizome ameliorates bilateral common carotid artery stenosis-induced cognitive deficit in mice by altering microglia and astrocyte activation
Source: Front Pharmacol. 2024 Mar 14;15:1329895. doi: 10.3389/fphar.2024.1329895 (PMC10973115; doi:10.3389/fphar.2024.1329895)
Supplement: Supplementary file 2 [file DataSheet1.DOCX]

**
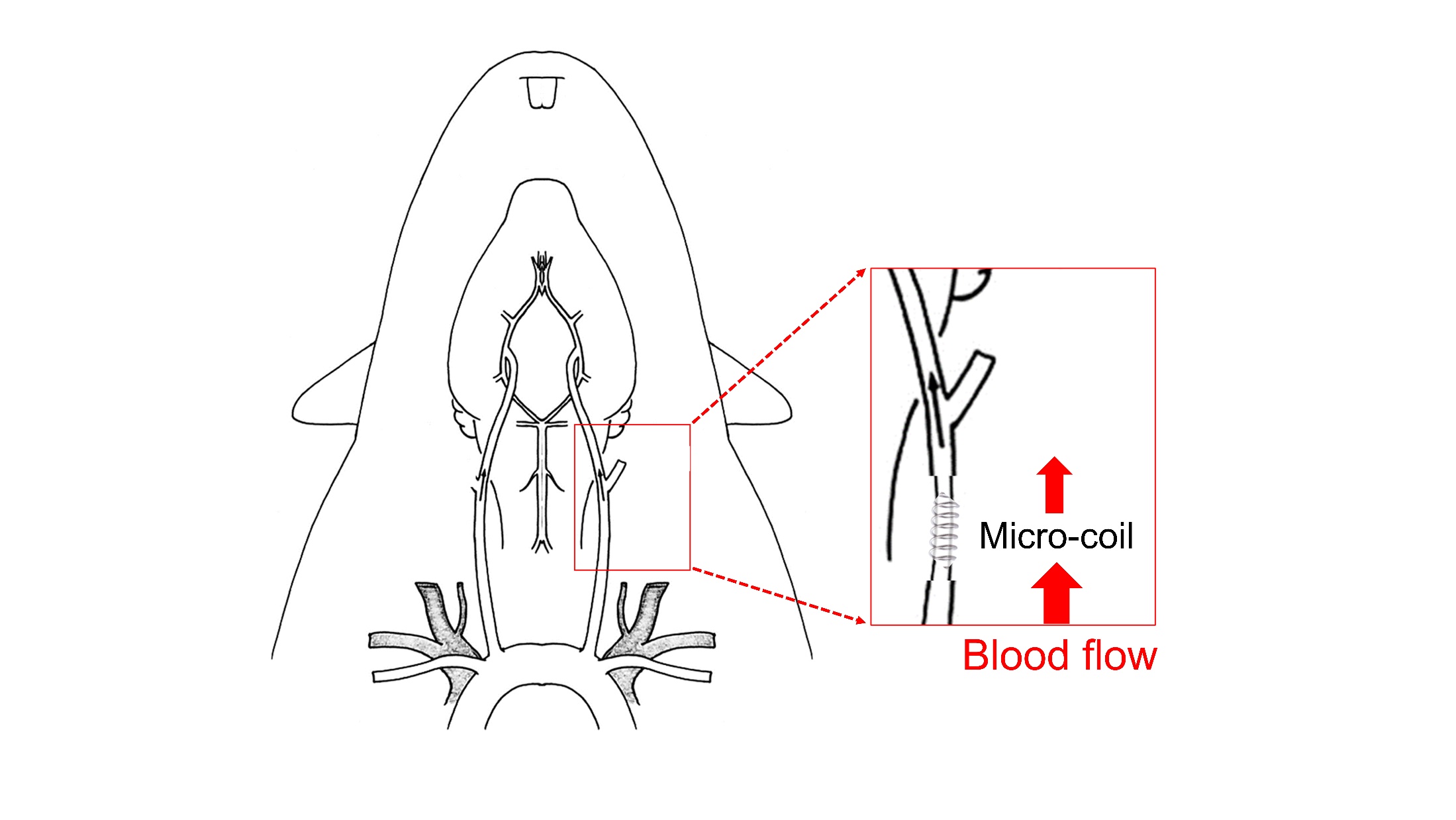
**

**Figure S1:** A schematic representation of BCAS using a micro-coil. Micro-coils with an inner diameter of 0.18 mm were wrapped around both carotid arteries to reduce blood flow to the brain, causing cognitive decline. This is a model used for mimicking human VaD in mice.


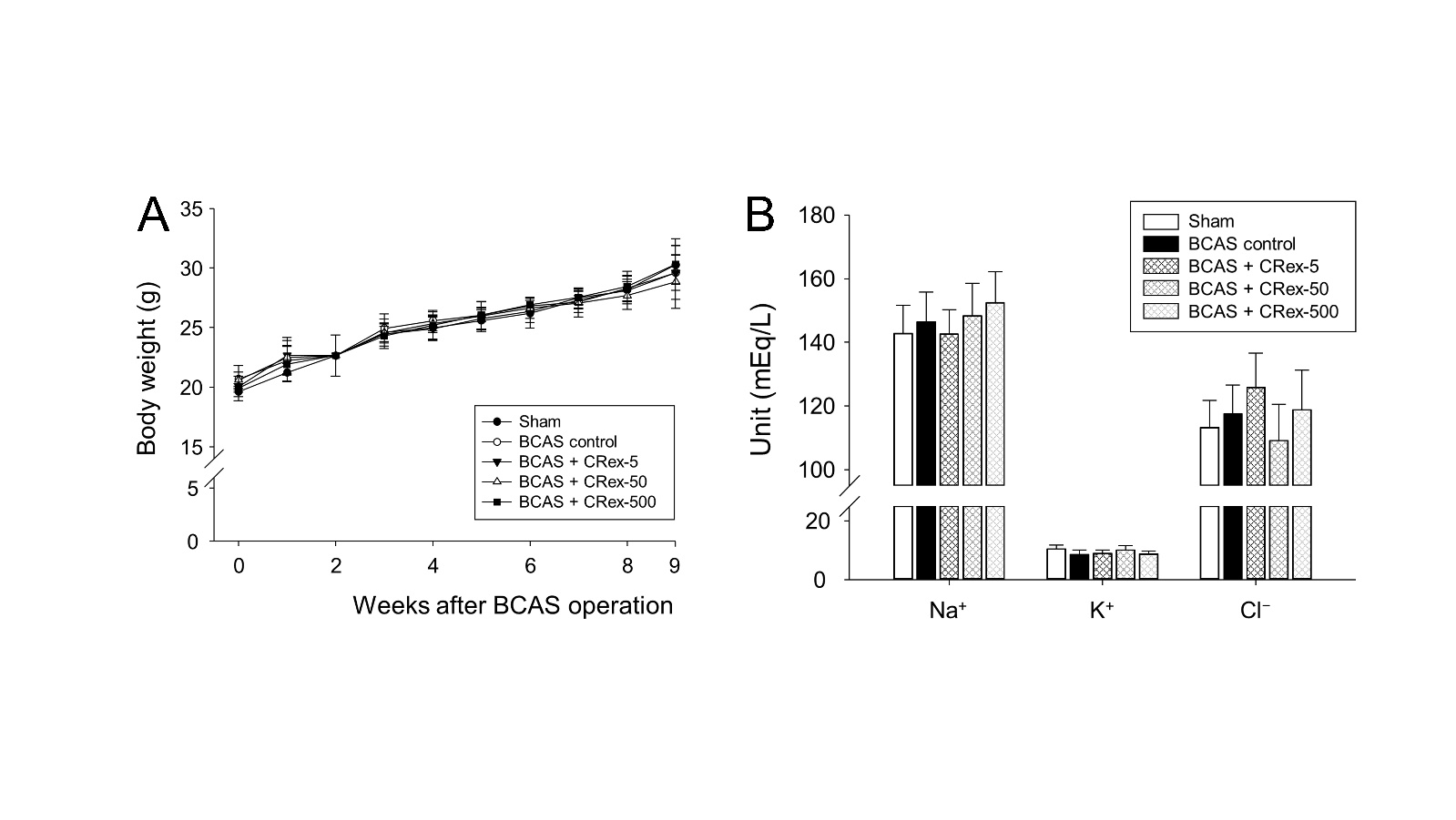


**Figure S2:** Changes in the body weight of mice during the experimental period and the electrolyte measurement values in blood at the end of the experiment. The body weight continued to increase even after surgery, with no significant difference between the experimental groups (A). No significant differences were observed between the experimental groups in terms of the values of electrolytes in the blood (B). The results are presented as mean ± SD.


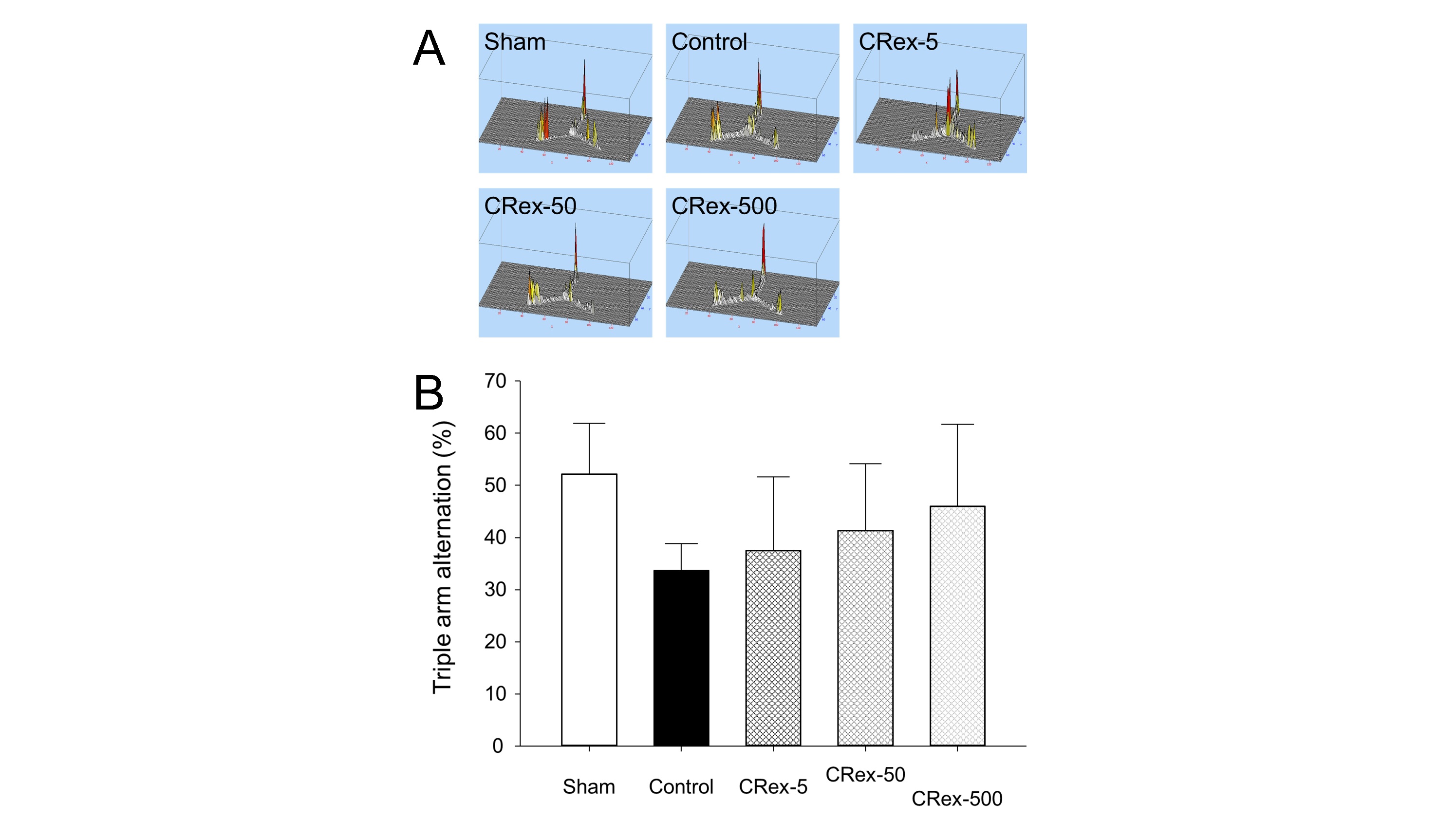


**Figure S3:** Triple arm alternation in Y-maze test. (A) The degree of movement and latency of the experimental animals in each arm of the maze determined visually. (B) The scores of sequential alternation in each arm of the maze. The results are presented as mean ± SD.


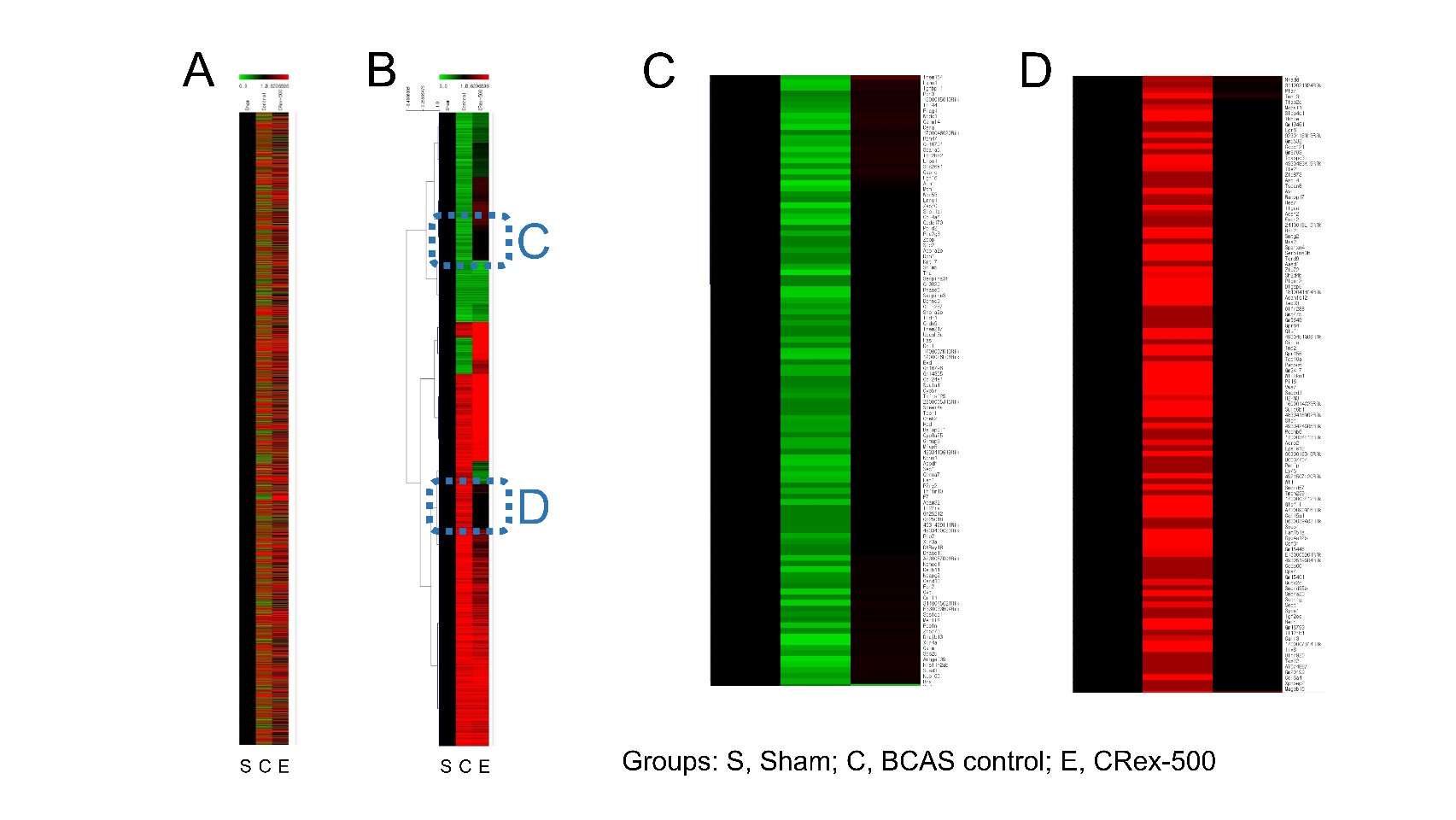


**Figure S4:** The clustering analysis of gene expression changes. The genes that increased or decreased in expression compared with Sham group were identified in each group. Red indicates genes that increased more than two-fold in expression, and green indicates genes that decreased to less than half in expression (A, B). C and D show some areas in which genes were regulated by administration of CRex.
